# Current management practices for patients presenting with low back pain to a large emergency department in Canada

**DOI:** 10.1186/s12891-017-1452-1

**Published:** 2017-02-23

**Authors:** Matthew L. Nunn, Jill A. Hayden, Kirk Magee

**Affiliations:** 1 Dalhousie Medical School, Dalhousie University, 5959 Spring Garden Road, Apt. 807, Halifax, Nova Scotia Canada; 2 Department of Community Health & Epidemiology, Dalhousie University, 5790 University Avenue, Room 403, Halifax, Nova Scotia B3H 1V7 Canada; 3 Department of Emergency Medicine, Charles V. Keating Emergency & Trauma Centre, Halifax, Nova Scotia Canada; 4 Department of Emergency Medicine, Dalhousie University, QEII HSC, Infirmary Site, 1796 Summer Street, Halifax, Nova Scotia B3H 3A7 Canada

**Keywords:** Low back pain, Emergency department, Management

## Abstract

**Background:**

Low back pain (LBP) is one of the leading causes of disability. Presentations to the emergency department (ED) are common and consume significant healthcare resources. However, treatment of patients with LBP is variable and highly physician dependent. Our study objective was to describe the demographic and clinical characteristics of patients presenting to the ED with LBP, the diagnostic strategies employed by ED physicians, and the subsequent management.

**Methods:**

We conducted a retrospective study using clinical and electronic health data at the Queen Elizabeth II Health Science Center’s Charles V. Keating Emergency and Trauma Centre. We selected a simple random sample of 325 adult participants who presented to the ED with non-urgent LBP over a six-year period. Data for all participants, including demographic characteristics, diagnostic testing, and interventions received, was retrieved from the Emergency Department Information System database and from patient charts.

**Results:**

Participants had a median age of 43 years and 55% were female. The majority (92.9%) were acute presentations of LBP (less than 4 weeks of duration), with an assigned Canadian Triage Acuity Scale score of 3-4 (92.4%). A range of pain intensity scores were reported, mostly without associated neurological symptoms (81%) or sciatica (68%). At triage, pain score was most commonly reported as moderate intensity (57.6%), followed by severe (32.6%) and mild (9.9%). Documentation of pain rating during assessment was similar (moderate 68.6%; severe 25.9%; mild 5.6%). Laboratory investigations were conducted on 22.5% of participants and 30% received an imaging study. Medications were delivered to 59.4% of participants during their stay in the ED. Of the medications administered, ibuprofen (28.3%), hydromorphone (24.9%), and acetaminophen (21.5%) were the most frequent. Almost all (94%) had a record of having a primary care provider in EDIS and referrals back to the participant’s family physician were recorded for 41.2% of non-urgent LBP encounters.

**Conclusions:**

We presented a complete description of patient characteristics, LBP descriptors, and health service use for a random sample of non-urgent LBP patients presenting to the ED. This has allowed for a better understanding of patients who seek care in the ED for their non-urgent LBP.

## Background

Low back pain (LBP) is very common worldwide, affecting people of all ages [1]. It is one of the leading causes of disability worldwide and has a high socioeconomic impact [1, 2]. A systematic review of the global prevalence of LBP estimates the point prevalence of activity-limiting LBP lasting more than 1-day to be 12%, with a 1-month prevalence of approximately 23% [3]. In 2016, an analysis for the Global Burden of Disease Study found back and neck pain is the leading cause of disability-adjusted life years (DALYs) in Canada and second overall in high income countries [4].

Low back pain has many potential etiologies; however, most patients (>85%) seen in primary care will have non-specific LBP and experience pain without an identifiable pathology or anatomical source [5]. Typically, non-specific LBP resolves within weeks without intense investigation or treatment [6]. Possible causes of LBP include vertebral compression fracture, radiculopathy, sciatica, and spinal stenosis, which occur in less than 10% of primary care patients and are generally non-urgent cases [5].

It is common for patients to present to an emergency department (ED) for investigation and treatment of their LBP. A recent systematic review that included 21 studies reported that in standard ED settings, 4.4% (pooled prevalence estimate) of visits are for LBP [7]. In the United States (US), there are approximately 2.7 million annual visits to the ED for LBP [8]. In Nova Scotia, LBP is the most prevalent health condition reported by those aged 20 to 44 years, and is the primary reason for 3.2% of all adult presentations to the ED [7].

An extensive evidence base and multiple clinical guidelines exist for the diagnosis and treatment of LBP in primary care settings [6], much of which may be relevant in the ED setting. However, limited research is available about treatment of patients with LBP within EDs. Many consider the ED a costly setting to treat LBP [9]. Referrals to other services, repeat presentations, and the use of diagnostic tests are common in ED settings, consuming physician time and healthcare resources. However, it has not been established whether treating LBP in the ED adds to overall healthcare costs.

One study of diagnostic testing and treatment of LBP in US EDs found that diagnostic imaging studies were ordered in approximately one-third of all LBP patients, and laboratory testing of urine and blood in 18.8 and 9.7% of LBP patients, respectively [8]. We are not aware of any studies that have been conducted in Canada investigating management of LBP in the ED.

The objective of this study was to describe the diagnostic and therapeutic strategies employed by ED physicians in a representative sample of patients with non-urgent LBP patients presenting to a Canadian ED.

## Methods

### Study design and data sources

We conducted a retrospective cross-sectional analysis of six years of clinical data from the administrative Emergency Department Information System (EDIS) dataset of the Charles V. Keating (QEII HSC ED) Emergency and Trauma Centre, in Halifax, Nova Scotia, Canada, supplemented with data from linked patient charts. Data was collected between July 15, 2009 and July 15, 2015.

Halifax is the provincial capital of Nova Scotia and is a coastal city in eastern Canada. The QEII HSC ED, a tertiary care center, is the largest ED in Atlantic Canada. It typically serves 186 patients in a 24-hour period. Daily, there are approximately 30 ambulance arrivals and between 1-6 emergency helicopter arrivals. There is a total of 36 patient spaces (3 trauma rooms) in the ED and the department averages approximately 1.5 traumas a day. When arriving at the ED, patients are triaged based on presenting complaint and a limited physical exam with vitals is conducted. There are two treatment areas within the ED, the main ED and a minor treatment area where patients do not need to be monitored by nursing staff. Depending on the patient’s condition and triage level, they are seen by a physician in one of these areas. All patients presenting to the ED are seen, regardless of compliant. Low back pain accounts for approximately 2100 visits per year at the QEII HSC ED [7]. This study was reviewed and approved by the Nova Scotia Health Authority Research Ethics Board.

### Study population

We assessed management of non-urgent LBP for adults aged 16 and older (the minimum age of intake in the QEII HSC ED) presenting to the ED. We included patients with non-urgent LBP, defined by two subgroups – non-specific LBP and LBP with neurological signs or symptoms – using ICD-9 diagnostic codes (Appendix). Non-specific LBP was defined as localized pain, muscle tension, or stiffness without an identifiable, known pathology [10]. LBP with neurological signs or symptoms is attributable to spinal joints, discs, vertebrae, muscle or soft tissues. For this subgroup, the presence of radiculopathy and lumbar spinal stenosis were screened for in the review. Radiculopathy was defined as impairment of a nerve root, with symptoms of radiating pain, numbness, tingling, or signs of muscle weakness corresponding to a specific nerve root. Lumbar spinal stenosis was defined based on signs of neurogenic claudication, such as worsening of pain with activity and in certain postures, relieved by rest. Signs and symptoms of lumbar spinal stenosis included discomfort, sensory loss, and weakness in the legs, reflecting involvement of spinal nerve roots within the lumbar spinal canal. An additional pathologic cause LBP subgroup was described as LBP attributed to a specific, often serious cause, not primarily related to the back, such as abdominal aortic aneurysm, pancreatitis, pyelonephritis, or cancer. Patients receiving a diagnostic code compatible with pathologic cause LBP were excluded. In addition, we excluded patients deceased on arrival.

Using a computerized random number generator, we selected a simple random sample of 325 patients who presented to the ED with non-urgent LBP over the study period for this focused supplemental review. This sample size was determined to reflect variation in the population and specifically identify an adequate number of LBP patients who had LBP with neurological signs or symptoms; estimated to be approximately 10% of the non-urgent LBP population.

### Variables of interest

Information was extracted from the EDIS database for important participant factors including, but not limited to, age, gender, length of stay, triage coding, and diagnosis coding. The QEII HSC ED uses the Canadian Triage Acuity Scale (CTAS) for triage coding and ICD-9 for diagnosis coding. In Canada, CTAS is a five-level scale for classifying the acuity of a patient’s condition (1 = resuscitation, 2 = emergent, 3 = urgent, 4 = semi-urgent, and 5 = non-urgent) based on the presenting complaint. In terms of assessing pain severity in association with CTAS level, it considers the location, severity, and acuity to better predict life-threatening conditions [11]. The CTAS tool has been found to be a valid instrument for predicting admission rates, hospital length of stay, and diagnostic utilization [12]. The ICD-9 coding system is used in many facilities across the country and remains a high standard in disease coding. It was revised in 2013 by the National Centre for Health Statistics (NCHS) and the Centre for Medicare and Medicaid Services (CMMS) found in the US. An electronic data extraction tool was developed in MS Access to aid in a highly-targeted patient chart review. This provided data on variables that were not available through EDIS related to the care provided in the ED and discharge instructions.

We recorded information about diagnostic tests utilized in the ED to investigate non-urgent LBP, treatment either administered or prescribed, and additional care provided. Diagnostic testing included plain radiograph, CT, MRI, urinalysis, and bloodwork. Treatment strategies included pharmacological and non-pharmacological therapies delivered in the ED and any prescriptions at discharge. Pharmacologic interventions were grouped into the following drug categories: anti-inflammatories, analgesics, opioids, and muscle relaxants. Care provided included referral to family physician, referral to medical specialist, referral to physiotherapy, and a documented discharge or follow-up plan. Other variables included in the analysis described patient characteristics, LBP features, and the utilization of health services. Patient characteristics included age, sex, responsibility for payment, and whether the patient had a primary care provider. Low back pain was described by CTAS score, discharge diagnosis (using EDIS and chart), type of ED visit, presenting level of pain, duration of LBP complaint, and the presence or absence of sciatica and neurological symptoms. Presence of sciatica was defined as pain, numbness, tingling in the distribution of the sciatic nerve, radiating down the posterior or lateral aspect of the leg, usually to the foot or ankle. Presenting level of pain was captured in triage by EDIS and in the chart review independently. Both used an 11-point numerical rating scale for pain (0-10). Severity of pain was categorized based on this scale into mild (0-3), moderate (4-7), and severe (8-10) pain, the same groupings used in the revised CTAS guideline [11]. Duration of LBP complaint was recorded in weeks since the onset of low back pain and categorized as acute LBP (less than 4 weeks), subacute LBP (4 weeks to less than 12 weeks), or chronic LBP (greater than 12 weeks) [5]. Health service use measured method of arrival, arrival date and time, departure date and time, and departure destination.

### Statistical analysis

Data were analyzed using STATA IC 13.1 statistical software, including descriptive analyses and assessment of characteristics associated with outcomes. Categorical variables were described with frequencies (%). Continuous variables with a normal distribution were described with a mean value and standard deviation. If the data was not normally distributed, we used a median value with interquartile range in the analyses. Patterns of missing data were examined and described.

## Results

Sociodemographic and ED visit characteristics of the 325 participants were identified from the EDIS database and the linked patient chart (Table 1). Participants had a median age of 43 years, of which 179 were female (55%). Almost all (94%) had a record of having a primary care provider in EDIS. The majority of participants presented acutely (less than 4 weeks of duration), with a CTAS of 3-4 (less urgent, urgent). A range of pain intensity scores were reported. At triage, pain score was most commonly reported as moderate intensity (57.6%), followed by severe (32.6%) and mild (9.9%). Documentation of pain rating during assessment was similar, with 68.6% of participants reporting moderate intensity of pain, with 25.9% severe and 5.6% mild. Generally, presentations were without associated neurological symptoms (81%) or sciatica (68%). Of note, 21% of participants arrived by ambulance; median length of stay was 2.8 h.

**Table 1:** Patient, visit, and health service use characteristics of non-urgent LBP patients (*n* = 325)

Characteristics	Number (%)
Age, years (median, IQR)	43 (30–57)
Female, sex	179 (55.1)
Primary care provider	305 (93.9)
Type of ED visit (*n* = 272; 53 missing)	
Emergency	265 (81.5)
Direct to consult	4 (1.2)
811 referral	2 (0.6)
Return visit	1 (0.3)
Missing	53 (16.3)
CTAS score (1–5)	
2	18 (5.5)
3	124 (38.2)
4	176 (54.2)
5	7 (2.2)
Presenting pain severity EDIS (0–10) (*n* = 132; 193 missing)	
Mild (0–3)	13 (9.9%)
Moderate (4–7)	76 (57.6%)
Severe (8–10)	43 (32.6%)
Presenting pain severity Chart (0–10) (*n* = 54; 271 missing)	
Mild (0–3)	3 (5.6%)
Moderate (4–7)	37 (68.5%)
Severe (8–10)	14 (25.9%)
Duration of LBP complaint	
Acute (0–4 weeks)	302 (92.9)
Subacute (>4–12 weeks)	10 (3.1)
Chronic (>12 weeks)	13 (4.0)
Presence of sciatica (*n* = 219; 106 missing)	
Yes	70 (32.0)
No	149 (68.0)
Presence of neurological symptoms (*n* = 258; 67 missing)	
Yes	49 (19.0)
No	209 (81.0)
Responsibility for payment (*n* = 315; 10 missing)	
Department of Health, NS	261 (82.9)
Other Canadian Province	11 (3.5)
Self (Non-Canadian)	3 (1.0)
Worker’s Compensation Board, NS	28 (8.9)
Other	13 (4.1)
Method of Arrival (*n* = 317; 8 missing)	
Walk-in	251 (79.2)
EHS ground	66 (20.8)
Length of stay, Hours (median, IQR)	2.8 (1.92–4.43)
Departure destination	
Home	316 (97.2)
Admitted	8 (2.5)
Left against medical advice	1 (0.3)

In terms of the diagnostic strategies used by ED physicians at the QEII HSC ED for patients diagnosed with non-urgent LBP, 22.5% of participants received laboratory investigation, either urinalysis or blood work, and 30% received an imaging study (27% plain film x-ray) (Table 2).

**Table 2:** Frequency of diagnostic tests performed for non-urgent LBP patients at the QEII HSC ED (*n* = 325)

Diagnostic test	Number (%)
Any laboratory test	73 (22.5)
Urinalysis	71 (21.9)
Bloodwork	30 (9.2)
Any Imaging	96 (29.5)
Plain radiograph	89 (27.4)
CT	15 (4.6)
MRI	2 (0.6)
Plain radiograph and CT	11 (3.4)
CT and MRI	1 (0.3)
Only MRI	1 (0.3)

Pharmacologic intervention for treatment of LBP included medications delivered in the ED (Table 3) and prescriptions given to participants at time of discharge (Table 4). Medications were delivered to the majority of participants (59.4%) during their stay in the ED. Ibuprofen (28.3%), hydromorphone (24.9%), and acetaminophen (21.5%) were administered most frequently, with acetaminophen also being combined in analgesic medications with dual-action, such as acetaminophen/codeine (Tylenol 3), acetaminophen/oxycodone (Percocet), and acetaminophen/codeine/caffeine (Atasol). In addition, different classes of medications were given in combination for symptomatic relief. The pharmacologic classes most commonly combined were opioids, analgesics, and anti-inflammatory medications.

**Table 3:** Common medication classes delivered in the QEII HSC ED for non-urgent LBP patients (*n* = 325)^a^

Medication class	Frequent medication	Number (%)
Opioid		112 (34.5)
	Hydromorphone	81 (24.9)
	Morphine	16 (4.9)
	Acetaminophen and codeine (Tylenol 3)^b^	19 (5.9)
Anti-inflammatory		115 (35.4)
	Ibuprofen	92 (28.3)
	Ketorolac	27 (8.3)
Analgesic		90 (27.7)
	Acetaminophen	70 (21.5)
Muscle Relaxant		24 (7.4)
Antiemetic		12 (3.7)
Combination of Medications^c^		
Opioid and Anti-inflammatory		55 (16.9)
Opioid and Analgesic		51 (15.7)
Anti-inflammatory and Analgesic		50 (15.4)

^a^Frequent medications delivered within selected classes are also presented (>4.5%)

^b^Medications of dual-action (e.g. Tylenol 3) were counted in both medication classes (e.g. opioid and analgesic

^c^Combination of medications were separately delivered drugs

**Table 4:** Classes of prescription medications given at discharge to patients for LBP (*n* = 325)^a^

Medication class	Frequent medication	Number (%)
Opioid		125 (38.5)
	Hydromorphone	63 (19.4)
	Acetaminophen and codeine (Tylenol 3)^b^	47 (14.5)
Anti-inflammatory		66 (20.3)
	Ibuprofen	38 (11.7)
	Naproxen	25 (7.7)
Analgesic		77 (23.7)
	Acetaminophen	20 (6.2)
Muscle Relaxant		26 (8.0)
Combination of Medications^c^		
Opioid and Anti-inflammatory		29 (8.9)
Opioid and Analgesic		64 (19.7)
Anti-inflammatory and Analgesic		17 (5.2)

^a^Frequent medications delivered within selected classes are also presented (>4.5%)

^b^Medications of dual-action (e.g. Tylenol 3) were counted in both medication classes (e.g. opioid and analgesic

^c^Combination of medications were separately prescribed drugs

Prescriptions constituted all written orders on the patient chart for medications received by the participants for use following discharge. This did not include recommended use of over-the-counter medications, as this was analyzed separately. Overall, 55% of participants received a prescription, most commonly hydromorphone (19.4%), Tylenol 3 (14.5%), and ibuprofen (11.7%).

Participants also received non-pharmacologic care in the ED that was documented in the patient chart (Table 5). Specific advice for the use of heat (7.1%), ice (7.4%), or both (4.9%) for pain control were recorded, as well as, the recommended use of over-the-counter medications, as needed (33.9%). Advice was documented in 52.3% of participant encounters. Physicians also documented recommendations for physiotherapy, massage, and acupuncture and distributed patient education pamphlets. Participants received documented advice to exercise (3.1%), but more often to rest (10.8%).

**Table 5:** Recommended non-pharmacologic care interventions for LBP patients at the QEII HSC ED (*n* = 325)

Care provided	Number (%)
Heat	23 (7.1)
Ice	24 (7.4)
Heat and ice	16 (4.9)
Movement (including stretching)	14 (3.1)
Rest	35 (10.8)
Over-the-counter medication	110 (33.9)
Ibuprofen	85 (26.2)

Referrals back to the participant’s family physician were recorded in 41.2% of encounters and referrals to medical specialists were recorded for 11.2% of study participants. In-hospital referrals were uncommon (5.5%), with orthopedics, internal medicine, and neurosurgery being consulted (Table 6). At our center, orthopedics and neurosurgery alternate daily for spine call. Although not frequently documented, 28 participants (8.6%) were advised to seek physiotherapy without a formal referral. Overall, 301 participants (96.2%) had a documented discharge or follow-up plan (*n* = 312; 13 missing).

**Table 6:** Referrals to other hospital services or family physicians received by LBP patients (*n* = 325)

Referral	Number (%)
Family physician (*n* = 319; 6 missing)	132 (41.2)
Medical specialist (*n* = 322; 3 missing)	36 (11.2)
ED Hospital Referral	18 (5.5)

## Discussion

This is the first Canadian study to present information about characteristics of non-urgent LBP patients and the strategies used by physicians in the ED to manage their care.

Our study population is similar to that of EDs in the US. In comparison to EDs in the US, a retrospective analysis of data from the National Hospital Ambulatory Medical Care Survey (NHAMCS) from 2002 to 2006 found similar presentation rates between males and females (51.2% female) with patients most commonly presenting with moderate or severe pain (30.6 and 54.2%, respectively) [8]. For this study, determining the duration of LBP and whether neurological symptoms were present was dependent on appropriate documentation of the patient encounter. Over 90% of participants presented acutely; however, these episodes of severe discomfort could have been in the setting of established, chronic LBP, which was not assessed for in this review. A current prospective study will aim to address the length of experienced LBP on presentation to the ED. Establishing this is important, especially when considering how guidelines for imaging and other testing are applied in the clinical setting. For example, these guidelines do not recommend imaging for acute LBP, in the absence of red flags [6, 13].

In terms of diagnostic imaging, the NHACMS found diagnostic imaging studies were performed on approximately one-third of all patients, with slightly higher rates of patients receiving plain radiographs (30.5% in NHACMS v. 27.4% our study) and undergoing CT or MRI investigations (6.1% received CT or MRI in NHACMS v. 4.9% our study). While our findings are comparable to the US, these rates suggest that imaging studies may be overused for non-urgent LBP, considering the frequency of acute presentations. According to the American College of Radiology (ACR), uncomplicated acute LBP and/or radiculopathy (less than 6 weeks) does not warrant any imaging studies and should be considered a benign, self-limited condition [14]. Similarly, in October 2016, Choosing Wisely Canada released new guidelines in agreement with the ACR, advising against low back imaging in patients with nontraumatic LBP. It is only in the case that malignancy or infection is clinically suspected from red flags or pathologic markers, or if there are severe, progressive neurological deficits, that diagnostic imaging and laboratory testing are indicated to guide management [6, 14].

Guidelines for pharmacologic therapy in the ED are generally consistent within primary care for the treatment of LBP. Therapeutic recommendations list acetaminophen and NSAIDs (nonsteroidal anti-inflammatory drugs) as first-line and second-line agents, respectively [13]. In addition, other medications used as third-line therapies include opioids, muscle relaxants, and steroids. In our study, pharmacologic interventions were consistent with clinical guidelines, considering the severity of pain experienced in this patient population. Medications were administered to close to 60% of participants during their stay in the ED. Acetaminophen, NSAIDs, and opioids were used most frequently. Combination therapy was often used, although effectiveness studies on this practice are limited [8]. In the US, medications either administered in the ED or prescribed at discharge most frequently were opioids (61.7%), NSAIDs (49.6%), and muscle relaxants (42.8%) [15]. In terms of medications delivered in the ED, patients were administered opioids more frequently in the US (61.7% v 34.5%) with hydromorphone also being the most commonly prescribed opioid (32.3%). NSAIDs were given to 49.5% of LBP patients and NSAIDs and skeletal muscle relaxants were most commonly combined (26.2%) in the US population [8]. It remains unanswered whether this population of LBP patients is different from those who seek care and treatment in other primary care settings and whether clinical guidelines, not specific to the ED, should be applied in this setting.

Although it was not captured in this study the number of patients that went on to receive non-pharmacologic therapies, such as physiotherapy, massage, and acupuncture, current reviews and practice guidelines outline their role in treatment of LBP [1, 16]. Guidelines vary in terms recommendations of non-pharmacologic therapy for acute LBP; however, an approach suggested is to consider these treatments in patients that do not respond to first-line care [1]. In the setting that response is not favorable to education, reassurance, and analgesia, or in chronic LBP, there may be an increased role for these therapies. The 2016 NICE guideline for LBP and sciatica recommends massage only as a part multimodal therapy and against the use of acupuncture for management of LBP [16].

From an ED perspective, LBP presentations consume time and healthcare resources. Diagnostic imaging, referrals, and interventions contribute to this, as do pre-hospital costs. As found by this study, one in five participants arrived by ambulance and on average had a length of stay of close to 3 h. Multiple studies have investigated why patients present to the ED with non-urgent medical conditions. Themes that emerge in patient interviews include a perceived need for urgent medical care, difficulty scheduling an appointment with their family physician, or the family physician being unavailable [17–19]. Although 94% of participants in our study had a record of a primary care provider, they potentially sought care in ED for these reasons. A UK study found that 86% of non-urgent presentations reported being aware of an alternative to the ED for care [18]. Expectations of ED-delivered health care may result in patients choosing to visit the ED rather than their family physician, as patients perceive that it is easier or more efficient to access diagnostic services in the ED [17]. Future studies are needed to determine patient prognosis and health service outcomes related to management of LBP in the ED setting.

### Strengths and limitations

Our study results should be interpreted in the context of study strengths and limitations. Strengths of our study include complete information about the care LBP participants received in the ED including imaging studies, laboratory tests, pharmacologic, and non-pharmacologic interventions. Using a combination of available EDIS data supplemented by a patient charts allowed for a better understanding of therapeutic intervention following diagnostic testing. We used strategies of data checking across ED charts and nursing records to limit potential measurement error: reports were compared to specific details about what each participant received, including dose and route of administration. A limitation of this study is that we did not examine care delivered to participants prior to arrival, for example, by paramedics. In addition, information gathered from the patient charts was limited by missing documentation of pertinent findings on history and physical examination. For example, presenting pain level was poorly documented. There were 193 missing records in EDIS (59.3%) and 271 in the medical chart (83.1%). In addition, noting the presence of neurologic symptoms and sciatica was dependent on documentation of the assessment. Prospective studies are needed to fully describe the characteristics and prognosis of patients presenting to the ED with LBP so that appropriate interventions can be planned.

## Conclusion

We presented a complete description of patient characteristics, LBP descriptors, and use of health services for a random sample of non-urgent LBP patients presenting to the ED. This facilitates a better understanding of the population of LBP patients who seek care in the ED and sets the stage for future studies to explore prognosis and test interventions appropriate for this setting.

## Appendix

**Table 7:** Coding system used to define LBP. Codes have been separated into three LBP categories: Non-specific, Mechanical and Non-Mechanical LBP. Two classes of LBP (urgent and non-urgent) were defined based on these categories. Corresponding ICD-9 codes are presented below

Non-Urgent (Non-Specific LBP and LBP with neurological signs or symptoms)		Urgent (Pathologic cause LBP)
Non-Specific LBP	LBP with neurological signs or symptoms	Pathologic cause LBP
•Backache •Back Sprain or Strain • Unspecified Back Disorder	• Compression Spinal Fracture • Degenerative Disc Disease • Disc Herniation • Discogenic • Scoliosis • Spinal Stenosis • Spondilolisthesis • Spondylosis • Unspecified Disc Disorders	• Abdominal pain. • Aortic Aneurysm • Cancer • Fracture of vertebral column • Headache / Migraine • Myalgia • Neuritis / Radiculitis • Pneumonia • Pancreatitis • Pyelonephritis • Renal Colic • Sciatica/ radiculopathy • Superficial Injury • Urinary Tract Infection
ICD-9: • 724 Unspecified back disorder • 724.2 Non specific etiologies (eg Lumbago) • 724.5 Backache, unspecified • 724.8 Other symptoms referable to back. • 729 other disorders of soft tissues • 846.9 Unspecified • 847 Sprain /strain back • 847.2 lumbar • 847.9 unspecified • 848 other and ill-defined sprains and strains. • 848.9 unspecified site. • 959 injury, other and un- specified. • 959.1 trunk injury. • 959.29 other site on trunk.	• 721.3 Spondylosis • 722 Intervertebral disc disorder • 722.1 Displacement of thoracic or lumbar intervertebral disc without myelopathy • 722.2 Displacement of intervertebral disc, site unspecified, without myelopathy • 722.52 Degenerative disc disease • 722.93 Other and unspecified disc disorder (Lumbar) • 724.0 Spinal Stenosis • 733.13 Compression fracture, not due to trauma. • 846 Sacroiliac sprains/strains • 846.0 Lumbosacral joint or ligament	• 140–239 Neoplasms • 346 Migraine • 441 Aortic Aneurysm • 486 Pneumonia • 577 Pancreatitis • 590.8 Pyelonephritis • 599 Urinary Tract Infection • 724.3 Sciatica • 724.4 Thoracic or lumbosacral neuritis/radiculitis • 729.1 Myalgia and myositis • 729.5 Pain in limb • 788 Renal Colic • 789 Abdominal Pain • 805 Fracture of vertebral column without mention of spinal cord Injury • 919 Superficial Injury

## Abbreviations

CMMS: Centre for Medicare and Medicaid Services
CTAS: Canadian Triage Acuity Scale
DALYs: Disability-adjusted life years
ED: Emergency department
EDIS: Emergency Department Information System
LBP: Low back pain
NCHS: National Centre for Health Statistics
NHAMCS: National Hospital Ambulatory Medical Care Survey
NSAIDs: Nonsteroidal anti-inflammatory drugs
QEII HSC ED: Queen Elizabeth II Health Science Center, Charles V. Keating Emergency and Trauma Centre
SDs: Standard deviations
US: United States of America
